# Anticipated Out‐Of‐Pocket Costs and Prostate Cancer Management Among Men With Commercial Insurance

**DOI:** 10.1002/cam4.71184

**Published:** 2025-08-29

**Authors:** Sarah Leick, Addison Shay, Samuel R. Kaufman, Xiu Liu, Paula Guro, Preeti Chachlani, Mary Oerline, Christopher Dall, Dawson C. Hill, Avinash Maganty, Vahakn B. Shahinian, Brent K. Hollenbeck, Arnav Srivastava

**Affiliations:** ^1^ Department of Urology Massachusetts General Hospital Boston Massachusetts USA; ^2^ Dow Division of Health Services Research, Department of Urology University of Michigan Ann Arbor Michigan USA

**Keywords:** financial toxicity, insurance design, out‐of‐pocket costs, prostate cancer

## Abstract

**Introduction:**

Men with newly diagnosed prostate cancer often appropriately elect for either immediate treatment or conservative management. The out‐of‐pocket costs they face vary by management strategy, with immediate treatment often superseding those of conservative management, potentially influencing patient decisions. We estimated the anticipated out‐of‐pocket costs that commercially insured men with newly diagnosed prostate cancer face and measured their association with immediate treatment.

**Methods:**

From MarketScan, we identified men with newly diagnosed prostate cancer from 2010–2020. Separately, using actual out‐of‐pocket costs (summing deductible, copay, coinsurance) among patients undergoing arthroscopic meniscal repair (*n* = 383,187), we derived regression coefficients for patient‐level variables (e.g., health plan type) that inform their financial liability. We applied these coefficients to men with prostate cancer and estimated their predicted out‐of‐pocket costs, our main exposure. We sorted patients into quartiles and used logistic regression to calculate adjusted probabilities of immediate treatment (versus conservative management).

**Results:**

We identified 58,206 men with prostate cancer and rank ordered them by predicted out‐of‐pocket cost. Approximately 12% of men had a predicted out‐of‐pocket cost of zero, and among those with non‐zero cost sharing, the median out‐of‐pocket cost was $350 (IQR: $275, $486). Across quartiles of predicted out‐of‐pocket costs, adjusted percentages of immediate treatment were in a narrow range between 77.8% (95% CI: 76.8%, 78.8%) for Quartile 1% and 78.6% (95% CI: 77.7%, 79.5%) for Quartile 4.

**Conclusion:**

Among commercially insured men with prostate cancer, predicted out‐of‐pocket costs varied substantially. However, the choice of management, immediate treatment or conservative management, appears insensitive (i.e., inelastic) to patient anticipated financial liability.

## 1 Introduction

Most men with newly diagnosed prostate cancer can be appropriately offered a broad spectrum of management strategies, from immediate treatment to conservative management [1]. However, it is often challenging to determine which patients should receive immediate treatment, even in the younger, healthier commercially insured population. On one hand, immediate treatment (i.e., surgery or radiation therapy) is curative in intent, but is often accompanied by substantial morbidity, including urinary, sexual, and bowel dysfunction [2]. On the other hand, conservative management strategies, such as active surveillance, allow patients to defer immediate treatment and its associated morbidity, but require periodic reassessment of cancer risk [3]. The range of prostate cancer management options available to most men introduces clinical uncertainty and underscores its discretionary nature, such that patient preferences often play a critical role in determining who elects for immediate treatment or conservative management [4].

Without definitive clinical consensus, the financial implications of healthcare decisions may drive patient preferences with regard to utilization [5, 6]. In prostate cancer, the out‐of‐pocket liability patients face across different management strategies varies substantially. Typically, the costs patients face for immediate treatment are greater than those for conservative management strategies [7, 8]. Further, rising out‐of‐pocket costs and their implications for quality of life magnify the financial tradeoff faced by patients with cancer [9, 10, 11, 12, 13]. Prior work in other clinical contexts demonstrates that this tradeoff influences patient behavior with respect to medication adherence for rheumatoid arthritis [14], taking preexposure prophylaxis for HIV [15], and treatment following wrist fracture [16]. Thus, anticipated out‐of‐pocket liability could influence management decisions in men with newly diagnosed prostate cancer, as in these clinical circumstances. Alternatively, a new diagnosis of cancer often evokes fear and anxiety in patients [17], which may make management decisions insensitive to the financial implications.

To study this, we empirically modeled the out‐of‐pocket liability patients face after a diagnosis of prostate cancer and measured its association with immediate treatment. We hypothesized that patients facing higher predicted out‐of‐pocket costs would have lower odds of undergoing immediate treatment, instead favoring conservative management.

## 2 Methods

### 2.1 Study Population

We identified men with private insurance and newly diagnosed prostate cancer between 2010 and 2020 using the IBM MarketScan Commercial Claims and Encounters Database. MarketScan contains deidentified inpatient, outpatient, and prescription claims data among patients with employer‐based health insurance. Incident prostate cancer diagnoses were identified using a previously established claims‐based algorithm, with a specificity of 99.8% and positive predictive value of 88.7% [18, 19]. Our cohort was limited to adult men under 65 years old, to ensure they did not have dual health insurance coverage under Medicare. We also required continuous coverage in the same health plan twelve months before to establish comorbidities [20], and twelve months after their prostate cancer diagnosis to assess management strategy.

### 2.2 Modeling Predicted Out‐Of‐Pocket Costs

Following a diagnosis of prostate cancer, the out‐of‐pocket liability a patient faces varies by management strategy (i.e., immediate treatment versus conservative management) [8]. In order to assess how a patient's anticipated out‐of‐pocket liability might influence decisions, the costs of a chosen management strategy must be weighed against the counterfactual of the patient electing for another option (i.e., what would a patient electing immediate treatment expect to pay if they had undergone conservative management or vice versa). These costs are likely influenced by patient‐level variables, such as age and health insurance plan type [21]. To characterize the effects of these patient‐level variables, we estimated an empirical model of out‐of‐pocket costs in men undergoing arthroscopic meniscal repair (*n* = 383,171).

Using arthroscopic meniscal repair to calculate the point estimates of these patient‐level variables allowed us to leverage the precision of one of the most common procedures among men with private insurance [21]. Actual out‐of‐pocket costs for arthroscopic meniscal repair were calculated by summing the deductible, copayment, and coinsurance. We then used a two‐part model—with a logistic regression (to account for patients with zero cost sharing) and a negative binomial regression (for patients with non‐zero cost sharing) component—to derive the underlying coefficients for patient‐level variables that inform out‐of‐pocket costs. These variables included age, geographical location (i.e., metropolitan statistical area introduced as a fixed effect), year of treatment, and health plan type. Importantly, health plans were classified as comprehensive coverage, high deductible, restricted network with non‐capitated payment (e.g., preferred provider organization), and restricted network with capitated payment (e.g., health maintenance organization). The large cohort of patients undergoing arthroscopic meniscal repair (versus the comparatively smaller cohort of patients with prostate cancer) allowed us to estimate precise coefficients across different combinations of patient‐level variables derived from our regression. These patient‐level coefficients were then applied to our cohort of men with newly diagnosed prostate cancer to estimate each patient's predicted out‐of‐pocket costs, which served as our main exposure and allowed us to rank order patients. We used this ranking of predicted out‐of‐pocket costs based on arthroscopic meniscal repair to reflect the ranking of patients by their anticipated costs for prostate cancer care (i.e., patients facing high costs for arthroscopic meniscal repair likely face high costs for prostate cancer care).

### 2.3 Statistical Analysis

Demographic variables and comorbidity data tabulated. We identified patients undergoing surgery or radiation therapy using claims‐based coding of procedure codes. We sorted patients into quartiles of increasing predicted out‐of‐pocket costs, with respect to their year of diagnosis. In this scenario, for example, when determining quartiles, men diagnosed in 2012 were compared against only other men diagnosed in 2012, not those in other years. Our study outcome, measured at the patient level, was the odds of undergoing immediate treatment (surgery or radiation therapy within one year of diagnosis) as opposed to conservative management. To assess the relationship between predicted out‐of‐pocket costs and odds of immediate treatment, we performed a logistic regression, adjusting for age, year of diagnosis, Charlson Comorbidity Index, and metropolitan statistical area (random effect).

### 2.4 Robustness Check

To confirm that our model could accurately rank order men by their predicted out‐of‐pocket costs, we compared quartiles of predicted out‐of‐pocket costs, derived through our methodology, against quartiles of actual out‐of‐pocket costs, calculated by summing deductible, copay, and coinsurance values from MarketScan. The resulting comparison demonstrated 68% agreement of quartiles. Additionally, the Cochran–Mantel–Haenszel test for stratum‐specific association demonstrated a statistically significant association between predicted and actual out‐of‐pocket costs across quartiles (*p* < 0.001).

All analyses were performed using SAS version 9.4 (Cary, NC) and STATA version 18 (College Station, TX). Average marginal effects (i.e., adjusted percentages) were calculated using postestimation commands (“margins” command). All statistical tests used *p* < 0.05 to define statistical significance. Our institutional review board deemed this study exempt from review.

## 3 Results

### 3.1 Cohort Characteristics

We identified 58,206 patients with newly diagnosed prostate cancer. As Table 1 illustrates, 53.0% (*n* = 30,832) were diagnosed between 2010 and 2013. This is consistent with the global decrease in MarketScan's population over time, not limited to prostate cancer diagnoses [9]. While age distribution was similar across quartiles of predicted out‐of‐pocket cost, men in Quartile 4 (i.e., highest costs) had a slightly lower comorbidity score distribution compared with men in Quartile 1 (CCI = 0 in 82.6% of men in Quartile 4 vs. 78.8% in Quartile 1, *p* < 0.001). Among patients in Quartile 1, most enrolled in restricted provider network plans with a capitated payment model (*n* = 7466, 51.0%) with relatively few patients enrolled in high‐deductible health plans (*n* = 299, 2.1%). Conversely, in Quartile 4, most patients were enrolled in a high‐deductible plan (*n* = 7542, 52.0%).

**TABLE 1:** Cohort characteristics by quartile of predicted out‐of‐pocket costs.

		Quartile 1	Quartile 2	Quartile 3	Quartile 4	*p* value*
N		14,588	14,534	14,584	14,500	
Age, mean (SD)		60 (4.6)	60 (4.6)	59 (4.3)	60 (4.2)	< 0.001
Year of diagnosis, *n* (%)	2010	2159 (14.8%)	2147 (14.8%)	2163 (14.8%)	2143 (14.8%)	N/A
	2011	2396 (16.4%)	2394 (16.5%)	2406 (16.5%)	2382 (16.4%)	
	2012	1669 (11.4%)	1670 (11.5%)	1673 (11.5%)	1661 (11.5%)	
	2013	1507 (10.3%)	1479 (10.2%)	1492 (10.2%)	1491 (10.3%)	
	2014	997 (6.8%)	1002 (6.9%)	990 (6.8%)	996 (6.9%)	
	2015	1015 (7.0%)	1015 (7.0%)	1016 (7.0%)	1013 (7.0%)	
	2016	858 (5.9%)	858 (5.9%)	858 (5.9%)	858 (5.9%)	
	2017	1088 (7.5%)	1080 (7.4%)	1085 (7.4%)	1082 (7.5%)	
	2018	1095 (7.5%)	1083 (7.5%)	1093 (7.5%)	1080 (7.5%)	
	2019	1038 (7.1%)	1039 (7.2%)	1036 (7.1%)	1037 (7.2%)	
	2020	766 (5.3%)	767 (5.3%)	772 (5.3%)	757 (5.2%)	
Charlson comorbidity index	0	11,497 (78.8%)	11,718 (80.6%)	11,846 (81.2%)	11,971 (82.3%)	< 0.001
	1	1904 (13.1%)	1778 (12.2%)	1711 (11.7%)	1666 (11.5%)	
	2	812 (5.6%)	709 (4.9%)	709 (4.9%)	627 (4.3%)	
	3+	375 (2.6%)	329 (2.3%)	318 (2.2%)	236 (1.6%)	
Health plan type, *n* (%)	Comprehensive	1537 (10.5%)	821 (5.7%)	189 (1.3%)	76 (0.5%)	< 0.001
	High deductible	299 (2.1%)	543 (3.7%)	1973 (13.5%)	7542 (52.0%)	
	Restricted provider, capitated payment	7446 (51.0%)	659 (4.5%)	47 (0.3%)	14 (0.1%)	
	Restricted provider, non‐capitated payment	4155 (28.5%)	12,010 (82.6%)	11,933 (81.8%)	6602 (45.5%)	
	Missing	1151 (7.9%)	501 (3.5%)	442 (3.0%)	266 (1.8%)	

^*^ *p* value: ANOVA for continuous variable, chi‐squared test for categorical variable. Quartiles decided within each year, thus *p* value for year of diagnosis is not applicable.

### 3.2 Out‐Of‐Pocket Costs and Prostate Cancer Management Strategy

**FIGURE 1:**
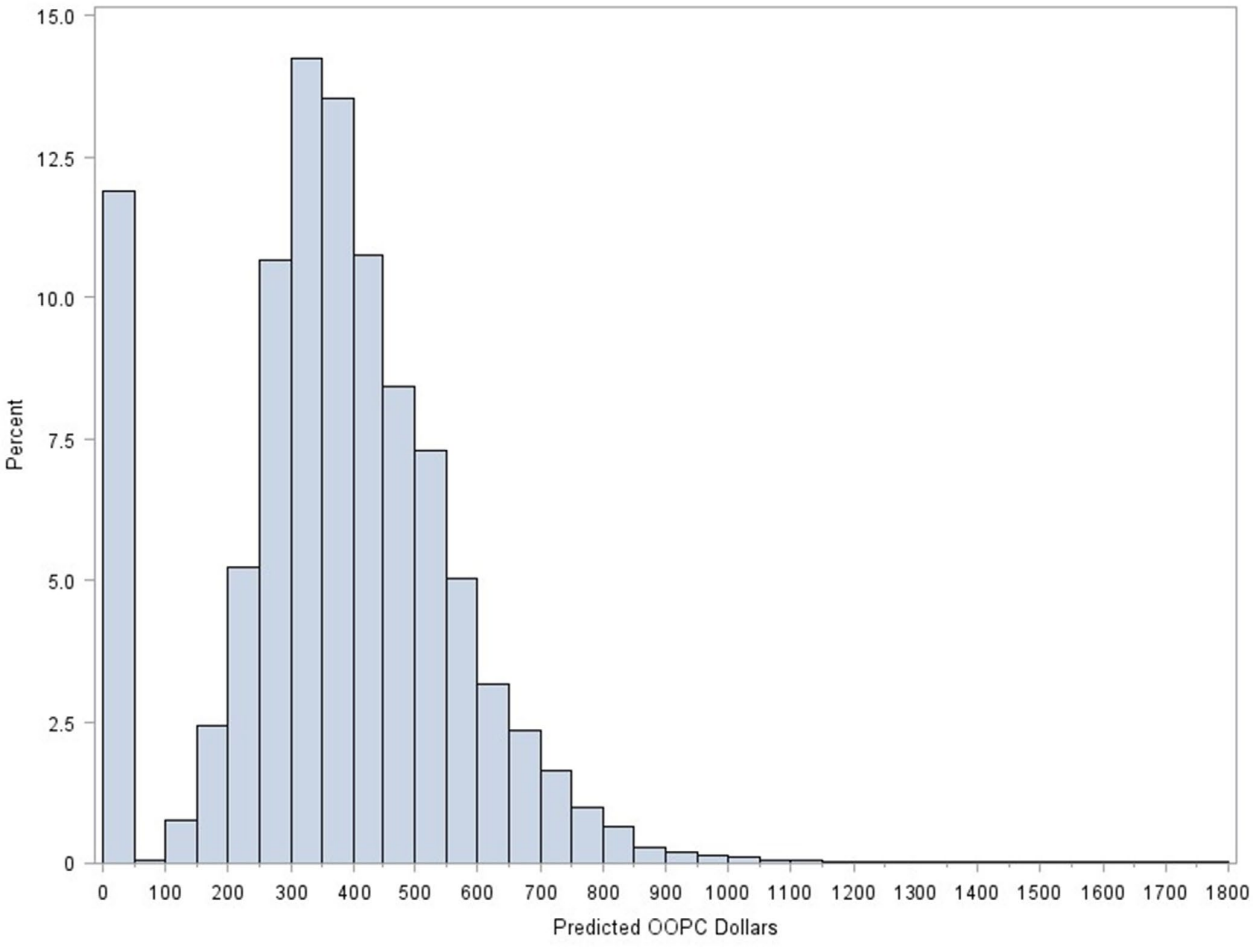
Distribution of predicted out‐of‐pocket costs based on arthroscopic meniscal repair among men with newly diagnosed prostate cancer.

**FIGURE 2:**
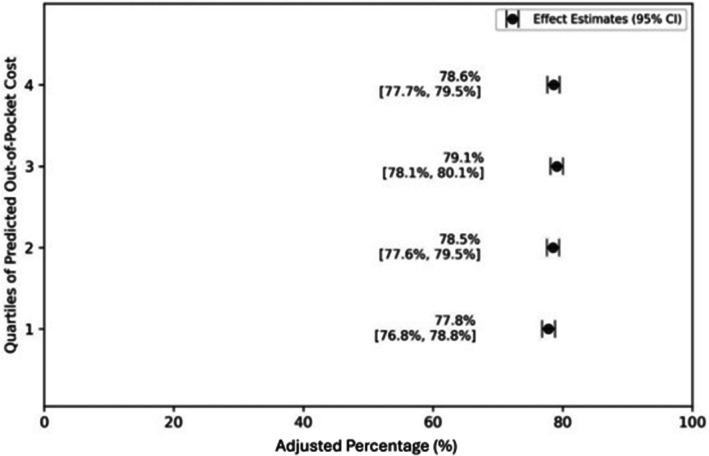
Adjusted* percentage of immediate treatment by predicted out‐of‐pocket cost. * *Adjusted for patient age, year of diagnosis, Charlson Comorbidity index, and metropolitan statistical area (random effect)*.

Figure 1 illustrates the distribution of predicted out‐of‐pocket costs among men with prostate cancer, based upon coefficients derived in the arthroscopic meniscal repair two‐part model (Table S1). Approximately 12% of men had a predicted out‐of‐pocket cost of zero. Among men with non‐zero costs, we observed a normal distribution with a median of $350 (IQR $275, $486). When modeling the odds of immediate treatment, men in Quartile 3 (adjusted OR 1.08; 95% CI: 1.01, 1.16; *p* = 0.02) were associated with greater odds than those in Quartile 1 (Table 2). We did not observe a statistically significant relationship between the odds of immediate treatment and men in Quartiles 2 and 4. As Figure 2 demonstrates, across all four quartiles, the adjusted percentage of immediate treatment was similar and had a narrow range between 77.8% (95% CI: 76.8%, 78.8%) for Quartile 1% and 79.1% (95% CI: 78.1%, 80.1%) for Quartile 3.

**TABLE 2:** Adjusted* odds of immediate treatment.

		Odds ratio	95% CI		*p* value
Quartile of predicted out‐of‐pocket costs	1	REF	REF	REF	REF
	2	1.04	0.98	1.11	0.17
	3	1.08	1.01	1.16	0.02
	4	1.05	0.98	1.13	0.19
Age (per year)		0.98	0.98	0.99	< 0.001
Year of diagnosis	2010	REF	REF	REF	REF
	2011	0.77	0.71	0.84	< 0.001
	2012	0.61	0.55	0.66	< 0.001
	2013	0.53	0.49	0.58	< 0.001
	2014	0.45	0.41	0.5	< 0.001
	2015	0.42	0.38	0.46	< 0.001
	2016	0.34	0.31	0.38	< 0.001
	2017	0.3	0.27	0.33	< 0.001
	2018	0.25	0.23	0.28	< 0.001
	2019	0.24	0.22	0.26	< 0.001
	2020	0.24	0.22	0.26	< 0.001
Charlson comorbidity index	0	REF	REF	REF	REF
	1	1.11	1.04	1.19	0.001
	2	1.04	0.95	1.14	0.38
	3+	1.15	1	1.32	0.04

* Also adjusted for metropolitan statistical area (random effect).

## 4 Discussion

In this study of men with private insurance, we examined how their anticipated financial liability after a prostate cancer diagnosis may influence management (i.e., immediate treatment versus conservative management). To do so, we modeled predicted out‐of‐pocket costs, leveraging the precision of a common surgical procedure (arthroscopic meniscal repair) exogenous to a patient's chosen management strategy. Based on our model, predicted out‐of‐pocket costs vary considerably across patients and are primarily driven by health insurance plan type. Patients in the highest quartile of predicted out‐of‐pocket costs most often enrolled in high‐deductible health plans. Overall, however, increasing predicted out‐of‐pocket costs did not significantly alter the odds of electing for immediate treatment.

Across a wide range of health services and indications, it is challenging to determine the price sensitivity of demand (i.e., elasticity) for healthcare utilization. Grouping various health services by global spending does not factor in the heterogeneity of healthcare necessity [22]. Prior work, however, has examined how anticipated costs may change patient behaviors in individual clinical contexts. Services with short‐term clinical implications and more predictable timeframes in which patients incur costs, such as maternity and emergency room care, appear relatively inelastic (i.e., utilization is not sensitive to the out‐of‐pocket costs patients face) [23, 24]. Conversely, where short‐term health effects are less clear or in scenarios where patients may accrue costs over a longer time horizon for unforeseen workups, patient demand for healthcare appears more elastic. For example, the use of preventative care services, including PSA‐based prostate cancer screening [25], typically decreases among patients enrolling in high‐deductible health plans compared with traditional health plans, which may buffer against these costs [26].

Along this spectrum, our results suggest that prostate cancer treatment is relatively inelastic with respect to predicted out‐of‐pocket costs among men with private insurance. While decisions around prostate cancer have been characterized as discretionary, there are at least two explanations for our findings. First, new cancer diagnoses are often accompanied by patient anxiety, which may override the cost of immediate treatment [17, 27]. Given this, despite the safety of conservative management in most patients with prostate cancer [28], they may view immediate treatment as more effective and deemphasize financial considerations [29]. Further, our study population consists of younger men who are most likely to benefit from immediate treatment and those with steady employer‐based private insurance, such that they are somewhat shielded from financial toxicity. As a result, this cohort may be relatively insensitive to cost considerations compared with patients in more tenuous financial circumstances.

Second, while shared decision making between patients and physicians is often referenced in clinical guidelines [30], urologist recommendation remains the dominant driver of a patient's management strategy [31]. This is evidenced by substantial practice‐level variation in the use of conservative management, not explained by differences in cancer risk characteristics [32, 33]. However, prior evidence suggests that physicians seldom discuss out‐of‐pocket costs with patients [34], particularly in the setting of cancer care, highlighting current shortcomings in shared decision making [35, 36, 37]. As a result, the primary factor in determining if a patient undergoes immediate treatment or conservative management—the physician recommendation—is often agnostic to the differences in costs between strategies and potential costs incurred by patients.

Our findings may point to current challenges in price transparency which prevent patients from adjusting their preferences to shield themselves from high out‐of‐pocket costs, even when less costly options are clinically appropriate. While our analyses take place prior to the Price Transparency Final Rule, enacted in 2021, patients' ability to estimate their out‐of‐pocket liability remains limited [38]. The ruling required hospitals to publicly post payer‐specific prices, standard charges, and discounted cash prices for at least 300 services. However, with relatively meager penalties (as little as $300 per day for smaller hospitals), over half of hospitals have not complied and reporting policies are typically haphazard [39, 40, 41]. For example, reporting prices for radical prostatectomy is mandatory, but optional for radiation therapy. As a result, in a study of National Cancer Institute‐designated cancer centers, only 10% of institutions report price estimates for external beam radiation therapy [42]. Even when prices are reported, most online transparency tools are poorly constructed and not user‐friendly, severely limiting patients' ability to price shop or make healthcare decisions which consider costs [43]. Policies aimed towards improving price information for patients, such as redesigning price transparency tools, may improve patient consumerism and prioritization of high‐value care.

Our study has several limitations. First, we predict out‐of‐pocket costs among men with prostate cancer by using coefficients derived from a model based on arthroscopic meniscal repair. Arthroscopic meniscal repair and prostate cancer treatment differ substantially as healthcare services. Consequently, underlying discrepancies in the costs and patient characteristics of these two cohorts may limit our ability to predict absolute costs and rank order patients by their anticipated out‐of‐pocket liability. However, we still observe nearly 70% agreement when comparing quartiles of predicted out‐of‐pocket costs and actual out‐of‐pocket costs among men with prostate cancer. Using arthroscopic meniscal repair allows us to leverage the precision of a more common procedure as well as develop construct validity for our model (e.g., greater out‐of‐pocket costs in men covered by high‐deductible health plans). Further, modeling out‐of‐pocket costs using a separate procedure makes our primary exposure exogenous to management decisions surrounding prostate cancer. Second, we do not have information regarding patient income or socioeconomic status. As a result, while we can classify predicted out‐of‐pocket costs, we cannot characterize financial toxicity. Placing these costs in the context of patient income may better contextualize the financial burdens patients face as well as longer‐term perceptions around their management decision [44]. Third, MarketScan claims data does not capture cancer risk or stage. These aspects of a patient's prostate cancer often inform management strategy, and thus we are unable to precisely determine if a patient would be appropriately managed with immediate treatment or conservative management. However, prior Surveillance Epidemiology and End Results Registry Medicare analyses demonstrate that prostate cancer risk is well balanced across practices with respect to management patterns [45]. Broadly, consistencies or differences in management strategy are unlikely to be explained by imbalances in cancer risk characteristics. Lastly, even within the categories of immediate treatment or conservative management, there may be substantial differences in utilization, which translate to differences in cost. For example, among patients undergoing conservative management, the use of expensive technologies such as magnetic resonance imaging and genomic testing may vary considerably [46].

## 5 Conclusions

Among commercially insured men newly diagnosed with prostate cancer, predicted out‐of‐pocket costs vary considerably and are heavily influenced by insurance plan type. Higher predicted out‐of‐pocket costs were not robustly associated with differences in patients electing for immediate treatment versus conservative management. Our work suggests, in the context of localized prostate cancer, that even when facing greater out‐of‐pocket liability, patients do not substantially change their cancer management preferences. Policymakers should consider improving price information accessibility to encourage prioritization of high‐value care.

## Author Contributions

**Sarah Leick:** conceptualization (equal), writing – original draft (lead). **Addison Shay:** formal analysis (lead), methodology (equal), writing – review and editing (equal). **Samuel R. Kaufman:** data curation (equal), formal analysis (equal), methodology (equal). **Xiu Liu:** formal analysis (equal), methodology (equal). **Paula Guro:** project administration (equal). **Preeti Chachlani:** investigation (equal), writing – review and editing (equal). **Mary Oerline:** data curation (equal), methodology (equal). **Christopher Dall:** writing – review and editing (equal). **Dawson C. Hill:** writing – review and editing (equal). **Avinash Maganty:** writing – review and editing (equal). **Vahakn B. Shahinian:** conceptualization (equal), funding acquisition (equal), project administration (equal), resources (equal), supervision (equal), writing – review and editing (equal). **Brent K. Hollenbeck:** conceptualization (equal), funding acquisition (equal), investigation (equal), methodology (equal), resources (equal), supervision (equal), writing – review and editing (equal). **Arnav Srivastava:** conceptualization (equal), formal analysis (equal), investigation (equal), methodology (equal), supervision (lead), writing – original draft (equal), writing – review and editing (lead).

## Conflicts of Interest

Arnav Srivastava reports grant funding from the National Cancer Institute. Brent Hollenbeck reports fees from Elsevier Publishing for professional activities and grant funding by the National Cancer Institute, National Institute on Aging, American Cancer Society, and Agency for Healthcare Research and Quality. Vahakn Shahinian reports grant funding by the National Cancer Institute, American Cancer Society, and Agency for Healthcare Research and Quality. No other authors have relevant conflicts of interest or disclosures.

## Supporting information


**Supplementary Table 1** Model to predict out‐of‐pocket costs among men undergoing arthroscopic meniscal repair

## Data Availability

The data underlying this article were provided by the IBM Marketscan Database and cannot be shared by the authors of this manuscript under the data use agreement.
