# Supplementary material for: Anticipated Out‐Of‐Pocket Costs and Prostate Cancer Management Among Men With Commercial Insurance
Source: Cancer Med. 2025 Aug 29;14(17):e71184. doi: 10.1002/cam4.71184 (PMC12395269; doi:10.1002/cam4.71184)
Supplement: Supplementary file 1 — Supplementary Table 1 Model to predict out‐of‐pocket costs among men undergoing arthroscopic meniscal repair [file CAM4-14-e71184-s001.docx]

**Supplemental Table 1: Model to predict out-of-pocket costs among men undergoing arthroscopic meniscal repair**

|  |  | *Logistic Model* | | | | *Negative Binomial Model* | | | | | |
| --- | --- | --- | --- | --- | --- | --- | --- | --- | --- | --- | --- |
|  |  | **Coefficient** | **95% CI** | | **P-value** | **Coefficient** | **95% CI** | | | **P-value** | |
| Age |  | 0.00 | 0.00 | 0.00 | <0.001 | 0.00 | 0.00 | 0.00 | 0.00 | |  |
| Health plan type | Comprehensive | REF | REF | REF | REF | REF | REF | REF | REF | |  |
|  | High deductible | 0.64 | 0.58 | 0.69 | <0.001 | 0.50 | 0.47 | 0.53 | <0.001 | |  |
|  | Restricted provider, capitated payment | -0.81 | -0.86 | -0.75 | <0.001 | -0.11 | -0.14 | -0.07 | <0.001 | |  |
|  | Restricted provider, non-capitated payment | 0.48 | 0.42 | 0.53 | <0.001 | 0.23 | 0.20 | 0.26 | <0.001 | |  |
|  | Missing | -0.19 | -0.25 | -0.12 | <0.001 | 0.22 | 0.19 | 0.26 | <0.001 | |  |
| Year of diagnosis | 2010 | REF | REF | REF | REF | REF | REF | REF | REF | |  |
|  | 2011 | 0.14 | 0.11 | 0.16 | <0.001 | 0.11 | 0.10 | 0.13 | <0.001 | |  |
|  | 2012 | 0.19 | 0.17 | 0.22 | <0.001 | 0.17 | 0.16 | 0.19 | <0.001 | |  |
|  | 2013 | 0.29 | 0.26 | 0.32 | <0.001 | 0.15 | 0.14 | 0.17 | <0.001 | |  |
|  | 2014 | 0.25 | 0.22 | 0.27 | <0.001 | 0.25 | 0.23 | 0.26 | <0.001 | |  |
|  | 2015 | 0.27 | 0.23 | 0.30 | <0.001 | 0.26 | 0.24 | 0.28 | <0.001 | |  |
|  | 2016 | 0.30 | 0.27 | 0.34 | <0.001 | 0.36 | 0.35 | 0.38 | <0.001 | |  |
|  | 2017 | 0.43 | 0.39 | 0.47 | <0.001 | 0.43 | 0.41 | 0.44 | <0.001 | |  |
|  | 2018 | 0.40 | 0.36 | 0.44 | <0.001 | 0.48 | 0.46 | 0.50 | <0.001 | |  |
|  | 2019 | 0.31 | 0.26 | 0.35 | <0.001 | 0.50 | 0.48 | 0.51 | <0.001 | |  |
|  | 2020 | 0.30 | 0.25 | 0.35 | <0.001 | 0.49 | 0.47 | 0.51 | <0.001 | |  |
